# The Under-Paid District Nurse

**Published:** 1921-03-05

**Authors:** 


### THE MATRONS' AND SISTERS' DEPARTMENT.

### THE UNDER-PAID DISTRICT NURSE.

The difficult problem of securing for trained nurses a salary commensurate with the economic value of their work cannot be tackled to any purpose without a study of the labour market. It is impossible to decide off-hand how much a nurse ought to earn without knowing what she can command in other directions.

The woman who aspires to be a district nurse gives her services at a small salary for three years in a general hospital, passes examinations, and then proceeds to give another six months to the practice of district nursing, also at a low salary, and (if she is to rank as fully qualified) puts in time at a tuberculosis dispensary or in a V.D. department, possibly both. The way to test her value to the public after taking this course of training is to consider what she could have earned in other directions before and after taking a course of special training extending over a period of three or four years. What salary could our candidate have commanded when, at the age of about twenty-one, she began her career? It is not necessary to exhaust possibilities in lay careers. It is sufficient to point to the fact that in nursing alone, quite as a novice, she could have earned from £47 to £50 a year in any one of the fever and miscellaneous institutions controlled by the Metropolitan Asylums Board, and about £70 in a mental hospital, in both cases inclusive of board, lodging, uniform, and laundry, with hours on duty restricted to fifty in the week and one whole day's leave once a week. It is idle to blame the M.A.B. and the mental hospitals for paying their probationers too high. They have to compete in the open market for labour and pay the market price, else they could not get their candidates at all.

But now compare the rate of pay which the highly-trained district nurse can command from her employers, the Nursing Associations, to whom she is indispensable.

The recently issued sixth annual report of the Central Council for District Nursing in London records: "The District Nursing Associations are unable to offer very attractive salaries, whilst at the same time demanding the highest qualifications." There was never a truer remark. For we find a few lines further on: "The Executive Committee have felt it right again to consider the minimum salary payable to district nurses by Associations in receipt of grants, and have increased this figure from £50 clear, over and above board and lodging, to £63, a sum which will not improbably need to be reconsidered in due course."

Now, the plain meaning of this is that, after passing through a rigid training at an unremunerative rate of pay for a period of close on four years, the district nurse, of whom "highest qualifications" are demanded, is worth to the public at best £13 more than she was as an untrained girl of nineteen or twenty, and at worst £6 less. The "highest qualifications" have, in fact, actually reduced her earning capacities.

There is no doubt that the rate of pay meted out to their nurses by the District Nursing Associations in London and other large towns is inadequate. It means this—that the nursing of the sick in the richest city in the world is to an overwhelming extent being carried out by the voluntary efforts of a devoted body of women who are forced to live as regards their own interests on the edge of a precipice. They are being deprived of the just fruit of their labours. They are relegated as regards salary to the position of totally untrained women, and we venture to prophesy that while this condition of affairs continues the Nursing Associations will continue to languish, not only as regards recruits for their service, but in the matter of subscriptions.

There is a forecast at the close of the report that a general appeal to the public is in prospect, in order that the voluntary associations may be adequately supported. We do not think that the Associations should appeal to the public until in this matter of just pay to their employees they have set their house in order. If the volume of work must be reduced let this be done forthwith. The nurses, no less than the patients, have bodies to be tended and kept in health. Let their hours be strictly limited to forty-eight or fifty per week. Let one day's rest in seven be a reality, provided for by a relay of emergency nurses, instead of a mockery dependent on the slight chance of no emergency case arising on a Sunday. Let the salary be fixed at the minimum suggested by the College of Nursing—£85 a year, plus board, lodging, uniform, and laundry. And throw on the great bulk of the patients the privilege they are in the majority of cases perfectly willing to assume of keeping the service in being.

The District Nursing Service is absolutely indispensable. The public can spare it as ill as they can spare the Private Nursing Service. But, unless it offers a better career than it does at present, it is certain that the best class of potential district nurses will be forced into other branches of work, and their priceless labours on behalf of their sick and suffering fellows will be lost to the community.

